# Harnessing Youths' Need to Contribute to Societal Challenges: A Naturalistic Experiment

**DOI:** 10.1002/jad.12517

**Published:** 2025-06-08

**Authors:** Lysanne W. te Brinke, Sophie W. Sweijen, Eveline A. Crone

**Affiliations:** ^1^ Department of Psychology, Education and Child Studies Erasmus School of Social and Behavioral Sciences, Erasmus University Rotterdam Rotterdam the Netherlands; ^2^ Faculty of Behavioral and Social Sciences Leiden University Leiden the Netherlands

**Keywords:** adolescence, civic engagement, intervention, mental health, societal contribution

## Abstract

**Introduction:**

Global societal challenges can negatively impact youths' mental health, but also be transformed into opportunities to have a positive impact. However, little is known about harnessing youths' need to contribute to societal challenges.

**Methods:**

We conducted a naturalistic experiment with 139 Dutch adolescents (*M*_age_ = 14.68, SD_age_ = 0.99, range = 12–17, 60.4% female). Adolescents in the experimental group participated in a societal challenge program, consisting of a workshop and impact challenge. Both the experimental and control group answered questionnaires about the need and perceived opportunities to contribute, feelings of vigor/depression, and novelty seeking at four time points (T1 = pretest, T2 = after the workshops, T3 = after the impact challenge, T4 = a 2.5‐month follow‐up).

**Results:**

There was an overall disbalance between adolescents' need to contribute and their perceived opportunities to make valuable contributions before the challenge program. This disbalance was enlarged for adolescents who were more sensitive to social rewards. After the workshops and impact challenge, the disbalance was smaller in the experimental group compared to the control group, and adolescents who participated with a higher challenge intensity, showed a higher need to contribute at T3. These differences were no longer observed at T4.

**Conclusions:**

Our findings provide initial evidence for the notion that some adolescents may be more inclined to become “agents of change” than others. By demonstrating that adolescents have a high need to contribute to societal challenges, this study provides important building blocks for research on adolescent mental health in a changing world.

## 1 Introduction

Adolescents who are growing up in the current decade need to deal with several societal challenges, such as increased social inequalities and climate change. These societal challenges do not only negatively impact youths' mental health (van Nieuwenhuizen et al. 2021), but can potentially also be transformed into opportunities for youth to have a positive impact. Scholars have suggested that the developmental phase of adolescence is characterized by an elevated need to contribute to society (Fuligni 2019). Initial empirical evidence suggests, however, that there might be a disbalance between adolescents' need to contribute to societal challenges and their perceived opportunities to make valuable contributions, and that this disbalance is associated with psychological well‐being (te Brinke et al. 2022; Fuligni et al. 2022). Therefore, the primary aim of this study is to empirically test this disbalance hypothesis and examine how this disbalance is influenced by novelty seeking behavior and a societal challenge program.

What is already known about the potential disbalance between the need to contribute and perceived opportunities? A cross‐sectional study demonstrated that during the Covid‐19 pandemic, most (> 80%) participating adolescents and young adults indicated that they were motivated to raise their voice and to help elderly community members. However, only a minority (< 7%) of participants had the feeling that their contributions were of actual value to society (te Brinke et al. 2021; te Brinke et al. 2022). These findings are in line with research on civic engagement (Hope 2016; Wray‐Lake and Ballard 2023). For example, in a cross‐sectional study among Black 10–15‐year‐old adolescents, the percentage of participants who indicated that they had helped the sick or elderly (e.g., > 80%) or participated in a neighborhood improvement project (e.g., > 65%) was relatively high, whereas feelings of political efficacy were relatively low (Hope 2016). This highlights the importance of considering not only youths' willingness to contribute to close others and the larger society, but also their perceived opportunities to make valuable contributions.

Denying adolescents the opportunity to contribute may have a negative impact on their psychological well‐being (Wray‐Lake and Ballard 2023). Adolescents who experience devaluation of their contribution due to ethnicity and/or gender (i.e., not being giving the chance to help others due to ethnic background), experience more feelings of depression in comparison to adolescents who do not experience such devaluation of their contributions (Fuligni et al. 2022). In contrast, increases in community engagement during adolescence and young adulthood have been linked to decreases in feelings of depression (Wray‐Lake et al. 2019), and adolescents and young adults who experienced more opportunities to contribute to society during the Covid‐19 pandemic, experienced higher levels of life satisfaction (te Brinke et al. 2022). Thus, decreasing the disbalance between youths' need to contribute and their experienced opportunities, may have positive effects on youths' well‐being.

Previous research shows that intervention programs that focus on prosocial behaviors toward close others (i.e., acts of kindness) and the larger society (i.e., volunteering) indeed have positive effects, on both the participating adolescents and their environment (for a review see Crone et al. 2022). A meta‐analysis focusing on school‐based prosocial behavior interventions, lasting between 5 weeks to 2 years, showed moderate postintervention effects (Mesurado et al. 2019). Similarly, challenge‐ and action‐based interventions, in which adolescents address community issues during the school year, have been shown to enhance civic self‐efficacy (Ballard et al. 2016). Together, these findings suggest that such interventions can empower adolescents through fostering stronger beliefs in their ability to effect change, thereby potentially reducing the disbalance between adolescents' need to contribute and their perceived opportunities.

Therefore, this study tests (1) the disbalance hypothesis of need and opportunity to contribute in relation to novelty seeking behavior (te Brinke et al. 2022). Specifically, social reward sensitivity and sensation seeking are selected as novelty seeking behaviors because previous research shows that these aspects are positively related to societal contribution (i.e., civic engagement and prosocial behavior; Maples‐Keller et al. 2016; Morelli et al. 2018). Next, we explore (2) whether the disbalance between adolescents' need and perceived opportunities to contribute decreases following a challenge‐based program, including whether program effects (i.e., dose) influence the relative effects of societal challenge‐based interventions. Finally, we test (3) how the disbalance in need and opportunity relates to feelings of vigor and depression. This study was carried out during the second year of the Covid‐19 pandemic, a time were the need to contribute was expected to be elevated. Specifically, during the Covid‐19 pandemic, opportunities to contribute were restricted due to lockdowns and other restrictive measures (Wilf et al. 2023), whereas youth's need to contribute appeared to be elevated (te Brinke et al. 2022).

**Figure 1:**
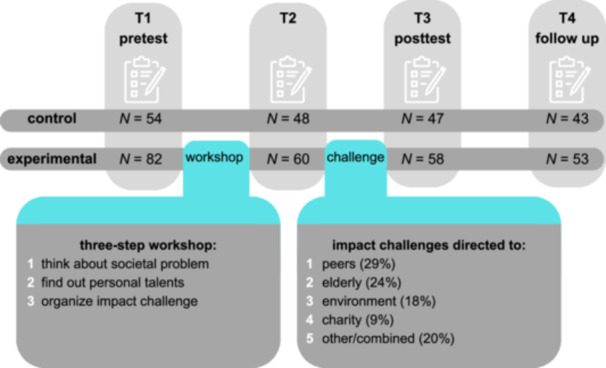
Overview of naturalistic experimental study design and loss to follow‐up.

We test these hypotheses in a naturalistic experimental study involving adolescents between ages 12 and 17‐year who participated in a societal challenge program (Figure 1). The age range was chosen to align with prior research (Hope 2016). We expect to show that adolescents will report a disbalance between need and opportunity (te Brinke et al. 2022; Fuligni 2019), with this effect being larger for high novelty seeking adolescents (te Brinke et al. 2022; Kam 2012). We also expect that the disbalance can be reduced by a societal challenge program (Mesurado et al. 2019), and we expect that a smaller disbalance is associated with higher well‐being (Wray‐Lake et al. 2019).

## 2 Materials and Methods

### 2.1 Participants

The study consisted of 139 Dutch adolescents between 12 and 17 years old (*M*_age_ = 14.68, SD_age_ = 0.99, 60.4% girl, 35.3% boy, 4.3% other or prefers not to disclose). In total, 84 adolescents participated in the societal challenge program (experimental condition). The other 55 participants did not follow the challenge program (control condition). Demographics for the total sample are displayed in Table 1. There were no significant differences between the two conditions in gender, birth country, religion, and level of family volunteering. Participants in the control (*M*_age_ = 14.92, SD_age_ = 0.85) condition were, however, significantly older than participants in the experimental (*M*_age_ = 14.53, SD = 1.05) condition, *F*(138) = 5.31, *p* = 0.023. Moreover, there were significant differences in SES composition between the control (low = 20.3%, middle = 54.1%, high = 25.7%) and experimental (low = 27.1%, middle = 29.2%, high = 43.8%) condition, *χ* ^2^(2) = 7.56, *p* = 0.023. Therefore, we controlled for age, gender and SES in all multigroup analyses.

**Table 1:** Demographic statistics of the study sample.

	Percentages (%)
Current education	
Prevocational education (VMBO)	53.3
Higher general continued education (HAVO)	20.7
Prepatory scientific education (VWO)	25.9
SES	
Low	23.0
Middle	44.3
High	32.8
Birth country	
The Netherlands	95.5
Other European country	2.9
Outside of Europe	1.5
Ethnicity	
Dutch	57.5
Moroccan or Moroccan‐Dutch	9.7
Turkish or Turkish‐Dutch	9.0
Surinamese or Surinamese‐Dutch	6.7
Antillean or Antillean‐Dutch	2.2
Polish or Polish‐Dutch	1.5
Other (e.g., multicultural)	13.4
Religion	
Nonreligious	60.3
Islamic	21.3
Christian	15.4
Hindu	2.9
Family volunteering	
Totally not common	15.3
Not common	35.8
Slightly common	38.0
Very common	10.9

### 2.2 Procedure

We conducted a naturalistic experiment, where online questionnaires were answered by adolescents who did (experimental group) or did not (control group) participate in a societal challenge program at their school (Figure 1). Adolescents from school classes across four different schools who signed up to participate in the societal challenge program received a general information letter of the study and consent form (experimental group). The schools chose which classrooms participated in the societal challenge program. We also asked the schools to distribute the information letter and consent form to a classroom that did not participate in the societal challenge program (control group) but was of a similar grade level as the participating classroom. After adolescents and the parents of adolescents who were younger than 16 years‐old signed informed consent, they received a link to the questionnaires. Specifically, questionnaires were sent before the societal challenge program (T1; pretest), at midpoint between the workshops and impact challenge (T2; after the workshops; on average 1 month after T1), after the impact challenge (T3; after the impact challenge; on average 1 month after T2), and at follow‐up (T4; on average 2.5 months after T3). Adolescents in the control group did not participate in the societal challenge program but were invited to answer the questionnaires at the same timepoints. Data collection took place between October 2021 and April 2022. The study was approved by the ethical committee of the Erasmus School of Social and Behavioral Sciences. Adolescents received €15,—compensation for their participation in the study.

#### 2.2.1 Societal Challenge Program

The societal challenge program was developed and provided by Young Impact, a Dutch foundation that helps youth to make impact and improve the world in a way that fits with their needs. Thus, the naturalistic intervention was already used in practice before the start of the research. The program consisted of two phases**:** (1) a workshop and information phase and (2) an impact challenge phase (Figure 1). In phase one, a three‐step workshop was provided by trainers from the Young Impact program and/or teachers. First, adolescents were encouraged to think about the societal problems that interested them most (i.e., climate change, loneliness of elderly people). Second, adolescents were encouraged to find out which personal talents (i.e., organizing, baking) they could use to contribute to this societal challenge. Third, adolescents were paired with classmates to organize an impact challenge of their choice. In phase two, adolescents were encouraged to perform their impact challenge (e.g., make a campaign to stimulate commuting by bike, baking cookies for elderly). Adolescents were supervised by their teachers in carrying out their impact challenge.

Participating adolescents could choose themselves on which societal challenge and target group they wanted to focus their impact challenge. Thus, the level of autonomy was relatively high. The impact challenges that were organized by the participants focused, for example, on peers (i.e., making a podcast to help peers with mental health problems), elderly (i.e., baking cookies and bring them to the nursery home), the environment (i.e., cleaning the streets), or a combination of target areas (i.e., organizing a campaign at school to stimulate peers to commute by bicycle or shared car, as this is better for both health and climate). For an overview of impact challenges that were performed by participants, see Figure 1.

### 2.3 Measures

#### 2.3.1 Needs and Opportunities for Societal Contribution

A revised version of the General Contribution to Society scale (van de Groep et al. 2020) was used to measure the needs and perceived opportunities for societal contribution. This scale consists of 10 items and two subscales: needs for societal contribution (7 items; e.g., “It is important to me to contribute to society”) and perceived opportunities (3 items; e.g., “I have the feeling that I am of value to society”) (te Brinke et al. 2022). We included both subscales in the current study. Participants rated the items on a scale from 1 (*totally not agree*) to 7 (*totally agree*). Internal consistency of the needs subscale ranged from *α* = 0.79 to 0.87, and of the perceived opportunities subscale from *α* = 0.62 to 0.73 across waves.

#### 2.3.2 Social Reward Sensitivity

The Prosocial Interaction subscale of the Social Reward Questionnaire for adolescents (SRQ‐A; Foulkes et al. 2014) was used to measure social reward sensitivity at T1. This scale includes 5 items (e.g., “I enjoy treating others fairly”) that are answered on a scale from 1 (*strongly disagree*) to 7 (*strongly agree*). Cronbach's *α* was 0.81.

#### 2.3.3 Sensation Seeking

The Dutch translation of the Brief Sensation‐Seeking Scale (te Brinke et al. 2022; Pechorro et al. 2018) was used to measure sensation seeking at T1. This scale consists of 8 items (e.g., “I would love to have new and exciting experiences, even if they are illegal”) that are answered on a scale from 1 (*strongly disagree*) to 5 (*strongly agree*). Cronbach's *α* was 0.70.

#### 2.3.4 Challenge Intensity

At T3, adolescents in the experimental condition were asked to describe the challenge that they performed (e.g., where, with what goal, and how). The answers to these open‐ended questions were coded on challenge intensity by a coder (i.e., research assistant), based on the challenge duration, degree of effort, contact with communities, and location. A detailed coding protocol for the challenge intensity variable was developed. The coder was trained thoroughly to minimize biases and ensure consistency throughout the coding process. The coder and first author discussed potential discrepancies in codes during the training phase. In total, the answers of 40% of the participants could be coded on a scale from 1 (*low intensity*) to 4 (*high intensity*). To measure inter‐rater reliability, 38.2% of these answers were double‐coded by a second coder. The inter‐rate reliability was moderate (ICC = 0.68).

#### 2.3.5 Feelings of Vigor and Depression

The shortened Dutch translation of the Profile of Mood States Scale (POMS; Wald and Mellenbergh 1990) was used to measure feelings of vigor and depression at T1‐T4. The vigor subscale includes 5 items (e.g., “vigorous”) and the depression subscale includes 8 items (e.g., “hopeless”). Participants were instructed to indicate to what extent they felt that the descriptions represented their mood state on a 5‐point Likert scale ranging from 1 (*not at all*) to 5 (*extremely*). Cronbach's *α* ranged from 0.66–0.81 for vigor and from 0.91–0.93 for depression.

### 2.4 Analyses

First, to examine the disbalance hypothesis of need and opportunity and the relation with novelty seeking behavior at the start of the challenge program, *t*‐tests and regression analyses were performed in SPSS version 29.0. A disbalance score was computed by subtracting the (individual) mean score of perceived opportunities from the (individual) mean score of need to contribute. As such, a higher disbalance score reflected a larger difference between the need to contribute and perceived opportunities. A paired t‐test was used to examine whether adolescents' overall need to contribute was significantly higher than their perceived opportunities to make valuable contributions. Regression analyses were performed with reward sensitivity/sensation seeking as independent variables and the disbalance scores as dependent variables to examine the association between novelty seeking behaviors and the disbalance between need and opportunity to contribute.

Second, to examine whether the disbalance between adolescents' need and perceived opportunities to contribute decreases following a challenge‐based program, four repeated measures ANCOVAs were performed. As this naturalistic study resulted in data loss at several time points (see Figure 1), analyses were performed per time point to include as many participants in each analysis as possible. In model 1, the change from T1 to T2 was examined, with the disbalance score as within‐person variable, condition (naturalistic experiment vs. control) as between‐person variable, and SES, age, and gender as covariates. In model 2, the change from T1 to T3 was examined, without taking T2 into account. In model 3, T2 was added as within‐person variable to examine change from T1 to T3. In model 4, T4 was added as within‐person variable to examine change from T1 to T4.

Third, to examine the effect of challenge intensity, hierarchical linear regression analyses with the difference score at T3 and T4 as dependent variable, SES, gender, age, and the difference score at T1 as independent variables (step 1) and the challenge intensity score as predictor (step 2) were performed.

Fourth, to examine how the disbalance in need and opportunity relates to feelings of vigor and depression, linear regression analyses were performed with disbalance scores as independent variables and feelings of vigor/depression as dependent variables. Lastly, changes in feelings of vigor and depression after the challenge program were examined with repeated measures ANCOVAs.

To account for clustering in the data on the school level, a fixed effect dummy variable approach was implemented for all repeated measure analyses. Specifically, we included dummy variables in the models (e.g., 3 school clusters, using the fourth school as reference category). This approach controls for unobserved heterogeneity within each cluster and allows for more accurate estimation of the effects of the predictors.

## 3 Results

### 3.1 The Disbalance Between the Need to Contribute and Perceived Opportunities

Descriptive statistics and correlations among study variables at the first timepoint (T1) are displayed in Table 2. Results of a *t*‐test showed that adolescents' overall need to contribute was significantly higher than their perceived opportunities to make valuable contributions, *t*(135) = 9.27, *p* < 0.001, *d *= 1.42 (Table 2). Thus, as expected, there was an overall disbalance (range −2.05 to 5.00) between adolescents' need to contribute and their perceived opportunities for societal contribution before the start of the societal challenge program. Follow‐up analyses showed that girls (*M* = 1.25, SD = 1.18) experienced a higher disbalance between the need to contribute and perceived opportunities than boys (*M* = 0.46, SD = 1.21; *F*(1,128) = 13.49, *p* < 0.001). Adolescents who did not identify as boy or girl (*N* = 6) also reported a disbalance (*M* = 1.63, SD = 0.97), but this subsample was too small to include in the analyses. No age effects on disbalance scores were found, *F*(1,134) = 0.22, *p *= 0.642.

**Table 2:** Mean scores and correlations between study variables at T1.

	Mean (SD)	1.	2.	3.	4.	5.	6.
1. Need to contribute	4.87 (1.05)	—					
2. Perceived opportunities to contribute	3.88 (1.37)	0.50**	—				
4. Disbalance need vs. opportunities	0.99 (1.24)	0.30**	−0.68**	—			
4. Reward sensitivity	5.96 (1.01)	0.49**	0.14	0.25**	—		
5. Sensation seeking	3.21 (0.73)	0.01	0.05	−0.05	0.03	—	
6. Feelings of vigor	3.67 (0.65)	0.06	0.10	−0.06	0.11	0.18	—
7. Feelings of depression	1.76 (0.82)	0.00	−0.08	0.09	−0.09	0.04	−0.42**

** *p* < .001.

### 3.2 Associations With Reward Sensitivity and Sensation Seeking

**Figure 2:**
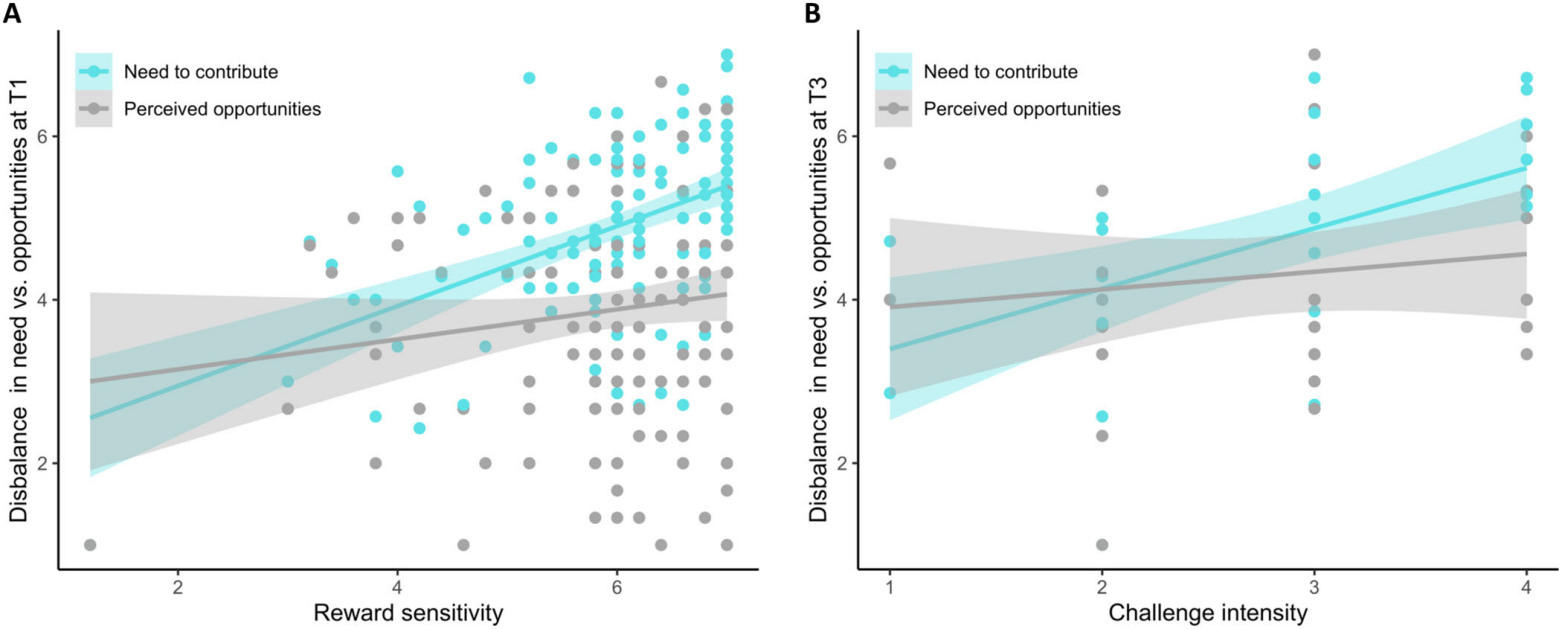
Associations between reward sensitivity and disbalance scores at T1 (A), and between challenge intensity and disbalance scores at T3 (B).

Next, we tested whether higher reward sensitivity and sensation seeking tendencies were associated with a larger disbalance. Indeed, regression analyses showed a significant effect of reward sensitivity on the disbalance scores at T1, *F*(1,133) = 8.70, *p* = 0.004. Thus, adolescents who indicated to be more sensitive to social rewards, experienced a higher disbalance between the need to contribute and perceived opportunities. Follow‐up analyses showed that this effect was mostly driven by the need to contribute (Figure 2A). There was a positive association between reward sensitivity and the need to contribute (*b* = 0.49, SE = 0.08, *β* = 0.49, *p* < 0.001), whereas the association between reward sensitivity and perceived opportunities was not significant (*b* = 0.18, SE = 0.11, β = 0.14, *p* = 0.107). No effects were observed for sensation seeking, T1, *F*(1,132) = 0.27, *p* = 0.603.

### 3.3 Changes in Disbalance Scores Following a Challenge‐Based Program

**Figure 3:**
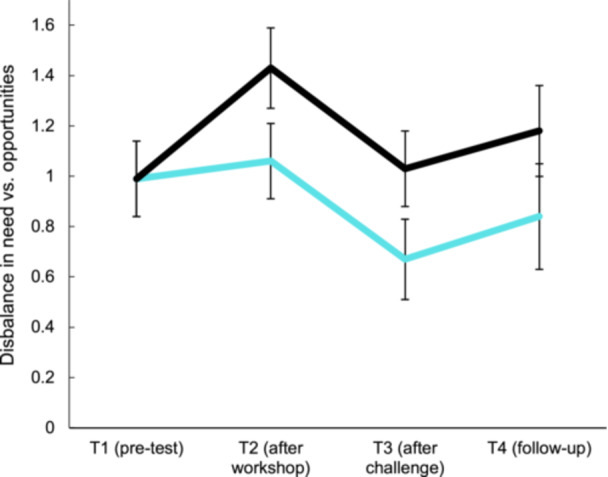
Effects of the societal challenge program on the disbalance between adolescents' need to contribute and their perceived opportunities from T1 to T4, with higher scores indicating a larger disbalance.

Descriptive statistics for the need to contribute, perceived opportunities, and disbalance between need and perceived opportunities at each timepoint (T1–T4) are displayed in the supporting materials (Table S1), as are the multivariate effects of all consecutive models (Table S2). A repeated measure ANCOVA with the disbalance score as within‐person variable (T1 = pretest, T2 = after workshops), condition (naturalistic experiment vs*.* control) as between‐person variable, and SES, age, gender, and the 3 cluster variables as covariates, showed no significant main effect of time on disbalance scores between T1–T2, *F*(1,88) = 0.40, *p* = 0.531, but there was a significant interaction between time and condition, *F*(1,88) = 5.29, *p* = 0.024. Examination of the estimated and observed means showed that adolescents in the experimental group reported a lower disbalance between their need to contribute and their perceived opportunities for societal contribution after following the societal challenge workshops at T2 (Figure 3). Follow‐up analyses showed that this effect was driven by opportunities to contribute. Specifically, there was a significant interaction between time and condition for opportunities to contribute between T1 and T2, *F*(1,88) = 4.17, *p* = 0.036, but not for the need to contribute, *F*(1,88) = 0.04, *p* = 0.850. When testing the change from T1 to T3 without including T2 in the analysis, the time by condition effect remained significant at T3, *F*(1,85) = 6.31, *p* = 0.014 (Figure 3). However, the difference between conditions in changes in disbalance scores was no longer significant when analyzing change from T1–T2 to T3 (after the challenge) and from T1–T2–T3 to T4 (follow‐up), as there was no significant interaction between time and condition from T1–T3 (*F*(2,75) = 2.79, *p* = 0.068) and from T1 to T4 (*F*(3,62) = 1.84, *p* = 0.345).

### 3.4 Program Effects: Challenge Intensity

A hierarchical regression analysis with the disbalance score at T3 (after the challenge) as dependent variable, SES, gender, age, and the difference score at T1 (step 1) and the challenge intensity score (step 2) as predictors, showed that challenge intensity predicted a significant proportion of the variance in disbalance scores at T3 in the experimental group, Δ*F*(1, 27) = 5.68, *p* = 0.024, Δ*R* ^2^ = 0.10. Regression coefficients are reported in Table 3. Follow‐up analyses showed that this effect was driven by the experienced need to contribute (Figure 2B). Specifically, adolescents who performed a more intense societal challenge, reported a higher need to contribute at T3, *b* = 0.40, SE = 0.14, *β* = 0.28, *p* = 0.007. In contrast, these adolescents did not experience more opportunities to contribute, *b* = −0.01, SE = 0.26, *β* = −0.01, *p* = 0.993. No such effect was observed at T4, Δ*F*(1, 18) = 0.08, *p* = 0.783 (see Table 3).

**Table 3:** Coefficients statistics for hierarchical regression analyses with SES, gender, age, the disbalance score at T1 (step 1) and the challenge intensity score (step 2) as predictors of T3 and T4 disbalance scores.

	Disbalance T3				Disbalance T4			
	*b*	SE	β	*p* value	*b*	SE	β	*p* value
*Step 1*								
Disbalance T1	0.47	0.14	0.49	0.002	0.39	0.31	0.29	0.219
Age	0.25	0.17	0.22	0.147	−0.04	0.30	−0.03	0.894
Gender	0.23	0.26	0.13	0.377	−0.41	0.44	−0.21	0.363
SES	0.46	0.23	0.28	0.058	0.94	0.43	0.44	0.042
*Step 2*								
Disbalance T1	0.46	0.13	0.13	0.001	0.36	0.33	0.27	0.283
Age	0.30	0.15	0.15	0.061	−0.03	0.32	−0.02	0.939
Gender	0.12	0.24	0.24	0.637	−0.37	0.47	−0.19	0.439
SES	0.26	0.23	0.23	0.275	0.98	0.47	0.46	0.050
Challenge intensity	0.44	0.18	0.18	0.024	−0.10	0.34	−0.06	0.783

### 3.5 Associations With Feelings of Vigor and Depression

Descriptive statistics for feelings of vigor and depression at each timepoint (T1–T4) are displayed in the supporting materials (Table S3). Regression analyses showed no significant effects of the disbalance scores on feelings of depression at T1, *F*(1,134) = 1.13, *p* = 0.219. Moreover, no significant effects of the disbalance score on feelings of vigor at T1 were found, *F*(1,134) = 0.45, *p* = 0.503. Thus, the disbalance between need to contribute and perceived opportunities was not related to feelings of vigor/depression. Lastly, repeated measure ANCOVAs showed that there were no significant changes over time in depression (T1‐T4; *F*(3,59) = 0.43, *p* = 0.731) and vigor (T1–T4; *F*(3,59) = 1.53, *p* = 0.216), or interactions between time and condition for depression (T1‐T4; *F*(3,59) = 0.37, *p* = 0.774) and vigor (T1–T4; *F*(3,59) = 0.74, *p* = 0.533). The multivariate effects are reported in the Supporting Information (Table S4).

### 3.6 Sensitivity Analysis

To account for attrition on the repeated measures, sensitivity analyses were performed. Little's MCAR test showed that the data were missing at random (*χ* ^2^(88) = 98.72, *p* = 0.204, Subsequently, we addressed missing data in BAM difference scores and feelings of vigor/depression over time using multiple imputation, generating 5 imputed data sets. Results were pooled to provide parameter estimates that account for uncertainty due to imputation, allowing us to retain cases with partially missing data. The findings are provided in the Supporting Information, Table S5.

## 4 Discussion and Conclusion

The current study tested whether there is a specific sensitivity in adolescence for opportunities to contribute to close others and the larger society, and which factors explain how adolescents can benefit from opportunities to contribute. These effects were tested with a naturalistic experimental challenge program during the Covid‐19 pandemic, which was considered to be a particular challenging time for adolescents in which they were given very few opportunities to contribute due to social restrictions (Wilf et al. 2023). We observed three important findings: (1) adolescents who show higher social reward sensitivity experience a larger disbalance between the need and perceived opportunities to make valuable contributions, (2) after the first and second phase of the societal challenge program (information, workshops, and societal challenge) this disbalance is smaller in the experimental group compared to the control group, and (3) adolescents who participated in a societal challenge program with a higher challenge intensity, show subsequently a higher need to contribute.

This study confirmed previous empirical and theoretical work (Fuligni 2019; te Brinke et al. 2022) by demonstrating that adolescents between ages 12–17‐year show a higher need than perceived opportunity to contribute. Thus, the need that adolescents have to raise their voice and help community members, is in disbalance with the degree to which they feel that they can make valuable contributions. We were therefore interested to examine which personality characteristics made adolescents more sensitive to experiencing this disbalance. We observed that social reward sensitivity was a strong predictor of the need to contribute, consistent with prior work showing that reward seeking behavior was predictive of prosocial risk‐taking (Blankenstein et al. 2020). Moreover, we observed that girls reported a higher disbalance between the need and perceived opportunities to contribute, consistent with prior work showing that girls report a higher need to contribute (te Brinke et al. 2022), more devaluation of contributions (Fuligni et al. 2022), and higher levels of social responsibility (Hope 2016) and prosociality (van der Graaff et al. 2018) than boys.

Interestingly, the disbalance in need and opportunity was not associated with well‐being as assessed in this study (feelings of vigor and depression), thereby suggesting that there may be different profiles of adolescents who feel a need to contribute and adolescents who suffer from negative feelings. Alternatively, the lack of association might be related to the age range of the sample (i.e., 12–17 years old). Early adolescence marks the beginning of political development where adolescents are not yet expected to fully participate in civic life (Wray‐Lake et al. 2019). Indeed, in previous research, the largest disbalance in need and opportunity to contribute was found in early adulthood (i.e., around 23–24‐year; te Brinke et al. 2022). Also, associations between opportunities to contribute and feelings of life satisfaction (te Brinke et al. 2022) and depression (Fuligni et al. 2022) were reported in the literature for somewhat older samples of youth. An important avenue for future research may therefore be to examine change trajectories of needs and opportunities in relation to mood trajectories across in the transition from adolescence to early adulthood.

The pandemic potentially resulted in two unique findings of this study. First, we observed that already after the first phase of the societal challenge program (workshops and information), adolescents in the experimental group showed a relatively smaller disbalance in need and opportunity (i.e., a stable effect) compared to adolescents in the control condition who showed an increasing disbalance, possibly associated with the ongoing pandemic where opportunities to contribute were limited (Wilf et al. 2023). The intention to contribute to close others and the larger society may potentially have triggered feelings of purpose, meaning and respect (Yeager et al. 2018). The next question was whether this disbalance further decreased after adolescents were provided the opportunity to perform an impact challenge (i.e., help peers or community members, raise their voice). We found partial evidence for this hypothesis, as the overall difference in disbalance between the experimental and control condition remained significant after the societal challenge. However, when testing the longer‐term effect (2.5‐month follow‐up), the disbalance was no longer significantly different between the experimental and control condition. On the one hand, the ongoing pandemic might have made it more difficult for adolescents to perform and/or finish their societal challenge. On the other hand, this lack of sustained effects may show that challenge‐based interventions have smaller long‐term effects, which is in line with previous research where mostly immediate intervention effects are reported (Mesurado et al. 2019).

This study is—to our knowledge—the first to examine follow‐up (e.g., 2.5‐month) effects of a school‐based impact‐challenge program. For example, earlier research with the Generation Citizen project, where adolescents choose a local issue to tackle collectively, learned strategies and skills for taking action, and developed and implemented an action plan, only examined direct effects (Ballard et al. 2016). The lack of sustained effects might be related to the relatively short duration and high level of autonomy that was provided in the current program to participating adolescents. This interpretation is in line with the notion that school‐based community engagement programs are more effective when they balance the level of supervision/guidance and autonomy (Simons and Cleary 2006). Alternatively, it is also possible that the lack of sustained effects of the societal challenge program is related to the relatively limited room that the program offered for self‐reflection (van Goethem et al. 2014).

Findings of the current study also pointed toward individual differences in the effects of school‐based societal challenge programs. Specifically, we observed that a more intense challenge experience (i.e., longer duration, more contact with community members) was associated with a larger need to contribute after finishing the impact challenge (i.e., at the third measurement point). Potentially, the freedom of choice that adolescence in the experimental condition received to choose their personal challenge contributed to this positive effect. This finding also suggests that some adolescents have the potential to become “agents of (societal) change,” as providing these adolescents with opportunities to contribute to a challenge that they find personally meaningful, resulted in an even higher need to contribute. This interpretation is in line with prior research on societal challenges, such as Black Lives Matter, showing that adolescents were highly engaged, and potential change makers within this movement (Baskin‐Sommers et al. 2021). Future research would benefit from revealing which individual factors make adolescents most committed to personally meaningful contribution goals, taking into account personal needs and opportunities.

This study has several limitations that should be taken into account when interpreting the findings of this study. First, due to the naturalistic nature of this study, we had relatively little control over the societal challenge program allocation and sampling. As a result, random assignment to the experimental and control group was not possible. Second, only 40% of participants provided detailed responses to the open‐ended question describing their challenge, which restricted the subset of data available for coding challenge intensity. Moreover, the naturalistic nature and limited information on the challenge, did not allow for a thorough investigation of program fidelity and compliance. Third, the lack of association between societal contributions and mental well‐being can potentially be explained by the included measurements. In the current study, mental well‐being was operationalized as feelings of vigor and depression, as opposed to more inclusive measures of well‐being (i.e., clinical depression and life satisfaction) that were used in previous studies on the association between need to contribute and adolescent mental health (te Brinke et al. 2022; Wray‐Lake et al. 2019).

Yet, it is also important to emphasize the unique nature of this study, conducted in a time of high societal need. The naturalistic nature of the study enabled us to assess the effects of a societal challenge program within youth's day‐to‐day educational contexts (Kember 2003). Taken together, this study confirmed the disbalance hypothesis, by demonstrating that adolescents who experience higher social reward sensitivity show a larger disbalance between their need and opportunities to make valuable contribution. The study further sheds light on the effects of programs that harness youth's need to contribute, by offering interpersonal and intersocietal prosocial experiences. This study therefore provides important building blocks for future research, by not only limiting our research focus to the potential adverse effects of 21st century societal challenges on adolescent mental health, but also on the positive effects of opportunities for youth to contribute to these societal challenges. As such, this study sheds important insight into our understanding of adolescent mental health in a changing world, such as the climate crisis (Thomaes et al. 2023) and social inequalities (Jamatia 2022).

## Ethics Statement

The study was approved by the ethical committee of the Erasmus School of Social and Behavioral Sciences. All adolescents and the parents of adolescents who were younger than 16 years‐old signed informed consent.

## Conflicts of Interest

The authors declare no conflicts of interest.

## Supporting information

Revised Supporting JAD 2024 0378r1.

## Data Availability

The materials, data, and code to reproduce all analyses in the manuscript are publicly available at the EUR Data Repository (http://10.25397/eur.24832269).
