# Supplementary material for: Harnessing Youths' Need to Contribute to Societal Challenges: A Naturalistic Experiment
Source: J Adolesc. 2025 Jun 8;97(6):1547–56. doi: 10.1002/jad.12517 (PMC12318459; doi:10.1002/jad.12517)
Supplement: Supplementary file 1 — Revised Supporting JAD 2024 0378r1. [file JAD-97-1547-s001.pdf]

Table S1

Descriptive Statistics for the Need, Perceived Opportunities for Societal Contribution, and Disbalance Between Need and Perceived Opportunities at Each Timepoint (T1-T4).

	Experimental Group		Control Group	
	M	*SD*	*M*	*SD*
Need T1	4.76	1.12	5.03	0.92
Need T2	4.87	1.30	5.25	0.93
Need T3	4.87	1.26	5.17	0.97
Need T4	4.94	1.11	5.08	0.81
Opportunity T1	3.77	1.49	4.04	1.14
Opportunity T2	3.81	1.29	3.81	1.09
Opportunity T3	4.20	1.33	4.14	0.91
Opportunity T4	4.10	1.48	4.31	1.08
Disbalance T1	0.99	1.35	0.99	1.24
Disbalance T2	1.06	1.19	1.43	1.17
Disbalance T3	0.67	1.16	1.03	1.10
Disbalance T4	0.84	1.53	1.18	1.38

Table S2

Multivariate Effects for Changes in Disbalance Scores from T1-T4 (complete data)

	Model 1: Time = T1 & T2 (df = 1, 88)		Model 2: Time = T1 & T3 (df = 1,85)	
	F	*p*	*F*	*p*
Time	0.40	.531	4.46	.038
Time*Condition	**5.29**	**.024**	6.31	.014
Time*Gender	2.06	.155	2.17	.144
Time*Age	0.05	.816	3.42	.068
Time*SES	0.04	.846	0.97	.329
Time*Cluster 1	0.01	.964	1.12	.294
Time*Cluster 2	0.14	.712	0.02	.877
Time*Cluster 3	1.65	.202	2.90	.092
	Model 3: Time = T1 – T3 (df = 2, 75)		Model 4: Time = T1 – T3 (df = 2, 75)	
Time	1.60	.210	2.28	.088
Time*Condition	2.79	.068	1.84	.345
Time*Gender	1.48	.235	1.60	.199
Time*Age	1.01	.368	1.90	.139
Time*SES	1.50	.230	1.13	.345
Time*Cluster 1	0.38	.686	1.71	.175
Time*Cluster 2	0.18	.836	1.05	.376
Time*Cluster 3	1.61	.207	1.16	.332

Table S3

Descriptive Statistics for Feelings of Vigor and Depression at Each Timepoint (T1-T4).

	Experimental Group		Control Group	
	M	*SD*	*M*	*SD*
Vigor T1	3.70	0.65	3.61	0.67
Vigor T2	3.46	0.69	3.44	0.71
Vigor T3	3.53	0.91	3.51	0.81
Vigor T4	3.52	0.78	3.57	0.65
Depression T1	1.74	0.81	1.79	0.82
Depression T2	2.01	1.03	1.86	0.95
Depression T3	1.97	1.00	1.98	0.97
Depression T4	2.24	0.99	1.96	0.92

Table S4

Multivariate Effects for Changes in Vigor and Depression Scores from T1-T4 (complete data)

	Vigor: Time = T1 – T4 (df = 3, 59)		Depression: Time = T1 – T4 (df = 3, 59)	
	F	*p*	*F*	*p*
Time	1.53	.216	0.43	.731
Time*Condition	0.74	.533	0.37	.774
Time*Gender	2.30	.086	1.39	.254
Time*Age	1.73	.171	0.31	.817
Time*SES	**3.02**	**.037**	1.84	.150
Time*Cluster 1	1.29	.285	0.20	.894
Time*Cluster 2	0.49	.691	0.08	.973
Time*Cluster 3	2.04	.118	0.20	.898

Table S5

Pooled Parameter Estimates for the 5 Imputed Datasets

	Experimental Group			Control Group		
	M	*SE*	*95% CI*	*M*	*SE*	*95% CI*
Disbalance T1	1.20	0.28	[0.65;1.75]	1.14	0.27	[0.27;2.00]
Disbalance T2	1.36	0.25	[0.87;1.85]	1.86	1.05	[1.05;2.67]
Disbalance T3	1.18	0.26	[0.66;1.69]	1.75	0.90	[0.90;2.60]
Disbalance T4	0.86	0.40	[0.59;1.67]	0.79	0.30	[0.13;1.87]
Vigor T1	3.50	0.15	[3.21;3.80]	3.35	0.24	[2.89;3.81]
Vigor T2	3.53	0.17	[3.11;3.78]	3.47	0.27	[2.95;4.00]
Vigor T3	3.58	0.22	[3.12;4.00]	3.55	0.37	[2.83;4.28]
Vigor T4	3.92	0.18	[3.33;4.04]	3.80	0.29	[3.22;4.37]
Depression T1	1.79	0.19	[1.42;2.16]	1.74	0.30	[1.16;2.33]
Depression T2	2.03	0.22	[1.60;2.46]	1.85	0.36	[1.13;2.57]
Depression T3	1.83	0.23	[1.38;.2.29]	1.57	0.37	[0.84;2.29]
Depression T4	2.12	0.25	[1.61;2.62]	1.83	0.38	[1.08;2.58]
